# Rare combined variations of renal, suprarenal, phrenic and accessory hepatic arteries

**DOI:** 10.1007/s00276-018-2026-0

**Published:** 2018-04-17

**Authors:** Łukasz Olewnik, Anna Waśniewska, Michał Polguj, Mirosław Topol

**Affiliations:** 1 Department of Normal and Clinical Anatomy, Interfaculty Chair of Anatomy and Histology, Medical University of Lodz, Lodz, Poland; 2 Department of Angiology, Interfaculty Chair of Anatomy and Histology, Medical University of Lodz, Lodz, Poland

**Keywords:** Common trunk, The accessory hepatic artery, The suprarenal artery, The inferior right phrenic artery

## Abstract

Knowledge of the morphological variations within the abdominal cavity is significant for all medical practitioners planning surgery. This report presents the rare origin of a common trunk for the right inferior phrenic artery, and superior and inferior suprarenal artery from the right renal artery. An accessory hepatic artery was found, which served as a branch of the right inferior phrenic artery. The diameter of the common trunk was 3.95 mm, and the diameters of the inferior and superior suprarenal arteries were 1.84 and 1.36 mm, respectively. The diameter of the right inferior phrenic artery was 2.55 mm. Both the embryological background and the potential clinical significance of this morphological variation are discussed. Knowledge of this common trunk and the occurrence of the accessory right hepatic artery may be of significance in diagnostic and surgical procedures.

## Introduction

The morphological variability existing between unpaired branches of the abdominal aorta has attracted the attention of anatomists, surgeons and radiologists due to its significance in a range of clinical procedures, including surgery of aneurysms or radiological transarterial chemoembolization procedures for tumours [19, 25, 26, 30].

A great deal of variation is seen in the origins of the inferior phrenic arteries (IPA), i.e. the right inferior phrenic artery (RIPA) and the left inferior phrenic artery (LIPA); however, they generally originate from the abdominal aorta or celiac trunk [1, 4, 10, 14–16, 21, 29, 32, 33], and are known to supply the diaphragm, oesophagus, stomach, liver, adrenal glands and retroperitoneum. The RIPA and LIPA may arise separately from the lateral aspect of the abdominal aorta, immediately above the celiac trunk, or by a common trunk. Some other variants may derive from the celiac trunk, the suprarenal, hepatic, left gastric, renal or superior mesenteric artery [1, 4, 10, 11, 14–16, 21, 25, 29, 32, 33].

The occurrence of accessory hepatic arteries (AHA), the left accessory hepatic artery (LAHA) and the right accessory hepatic artery (RAHA) have been described previously [12, 22–25, 30, 35].

Adrenal arteries (AA) supply the adrenal gland, and consist of three arteries: the superior suprarenal artery (SSA), the middle suprarenal artery (MSA) and the inferior suprarenal artery (ISA). The SSA arises from the LIPA, the MSA arises from the aorta between the IPA and the renal artery (RA), while the ISA derives from the RA; however, they may show variations in their origins [2, 7–9, 20, 21].

This case report describes a rare variant origin of a common trunk for the right inferior phrenic artery (which gives the right accessory hepatic artery) and the superior and inferior suprarenal artery from the right renal artery. Our findings highlight the importance of knowledge of the arterial supply in the abdominal cavity, and these findings are significant for radiologists, anatomists and surgeons specializing in the hepato-bilary and pancreatic areas.

## Case report

**Fig. 1:**
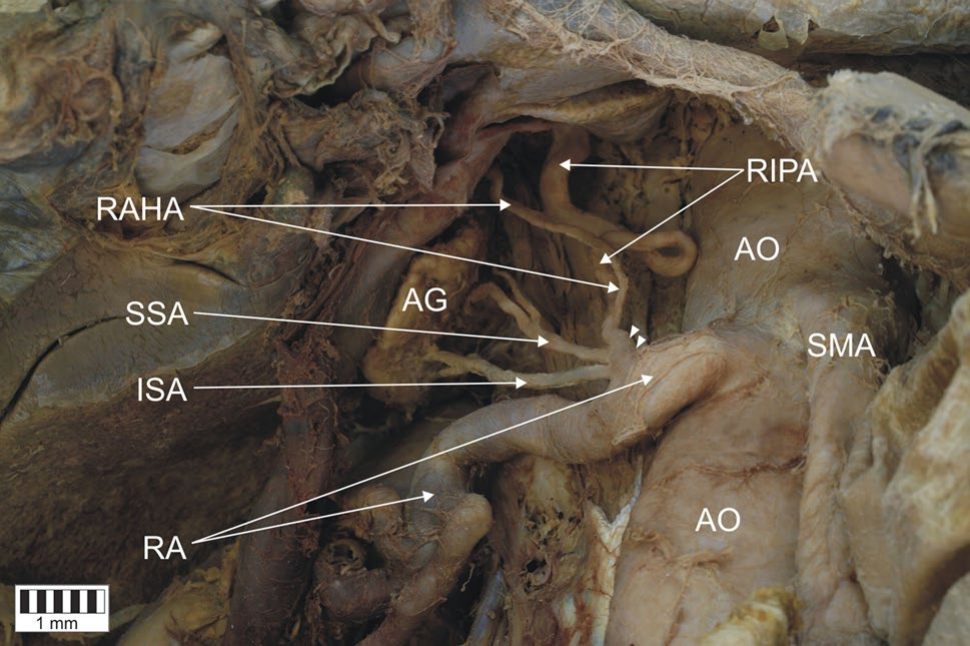
Origin of a common trunk for the right inferior phrenic artery (which gives rise to the right accessory hepatic artery) and inferior and superior suprarenal artery. White arrowheads present the common trunk for the right inferior phrenic artery, and inferior and superior suprarenal artery. *AO* abdominal aorta, *CT* celiac trunk, *SMA* superior mesenteric artery, *RIPA* right inferior phrenic artery, *RAHA* right accessory hepatic artery, *SSA* superior suprarenal artery, *ISA* inferior suprarenal artery, *RA* renal artery

**Fig. 2:**
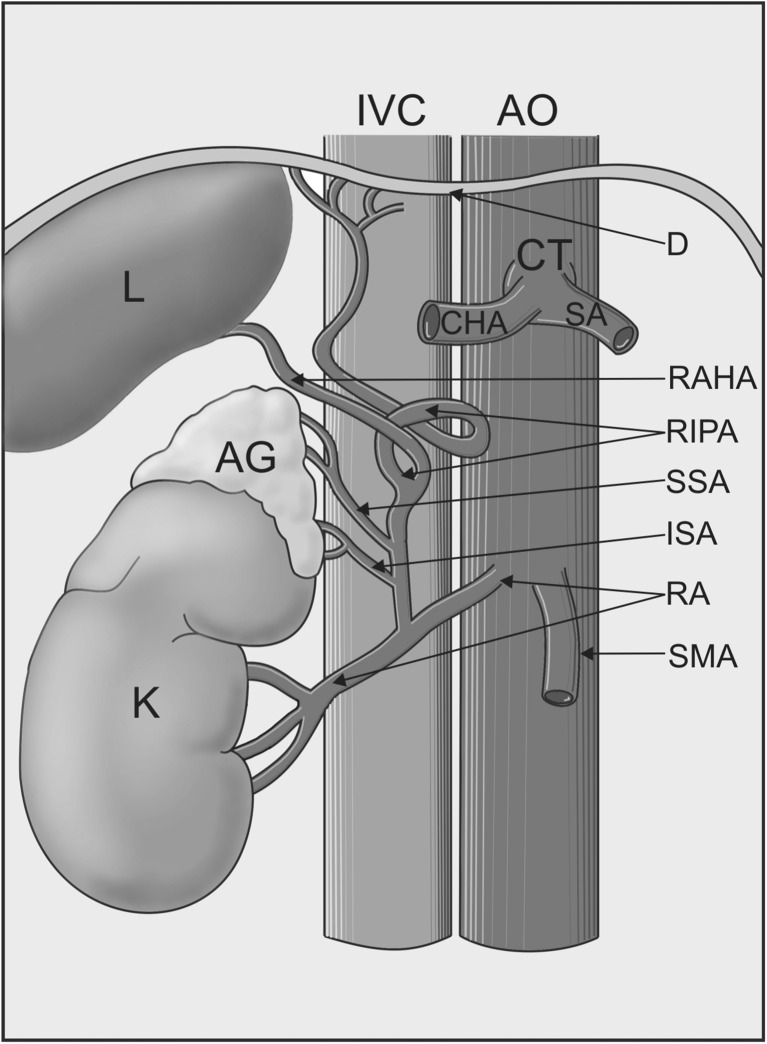
Schema of a common trunk for the right inferior phrenic artery (which gives the right accessory hepatic artery) and inferior and superior suprarenal artery. White arrowheads present the common trunk for the right inferior phrenic artery, and inferior and superior suprarenal artery. *AO* abdominal aorta, *SMA* superior mesenteric artery, *RIPA* right inferior phrenic artery, *RAHA* right accessory hepatic artery, *AG* adrenal gland, *SSA* superior suprarenal artery, *ISA* inferior suprarenal artery, *RA* renal artery, *IVC* inferior vena cava, *K* kidney, *L* liver, *D* diaphragm, *CT* celiac trunk, *CHA* common hepatic artery, *SA* splenic artery

The cadaver of a 64-year-old man was subjected to routine anatomical dissection for research and teaching purposes at the Department of Normal and Clinical Anatomy of the Medical University of Lodz. The dissection was performed in the abdominal cavity. A careful resection was performed of the interrupting tissues, where a common trunk of the right inferior phrenic artery, and superior and inferior suprarenal artery originated from the right renal artery, which originated from the anterior side of the abdominal aorta. The further course of the inferior right inferior phrenic artery gave rise to the right accessory hepatic artery (Figs. 1, 2). The right middle suprarenal artery was absent (Figs. 1, 2).

The arteries were measured using digital photographic documentation processed through MultiScanBase 18.03 (Computer Scanning System II, Warsaw, Poland). The value and precision of this method have been confirmed in a previous study [26, 31].

The diameter of the renal artery at its origin from the abdominal aorta was 10.03 mm (the diameter of the coeliac trunk was 12.84 mm, while the diameter of the superior mesenteric artery was 10.17 mm). After 12.53 mm, the right renal artery gave rise to a common trunk of the right inferior phrenic artery, and the superior and inferior suprarenal arteries. The diameter of the common trunk was 3.95 mm, the first branch was the inferior suprarenal artery (diameter 1.84 mm); following this, the course was divided into the superior suprarenal artery (diameter 1.36 mm) and the common trunk of the right inferior phrenic artery and the right accessory hepatic artery. The diameter of the right inferior phrenic artery was 2.55 mm. Furthermore, it was observed that the right hepatic artery branched out: where it did so, the diameter of the right hepatic artery was 1.13 mm and that of the right inferior phrenic artery was 2.11 mm.

## Discussion

Knowledge of the vascularization of hepatic vascular variations is significant for the daily practice for surgeons specializing in the hepato-bilary and pancreatic area, and also for general surgeons and radiologists, mainly those involved in interventional radiology. Significant improvements have been made in the surgical and/or radiological treatment of benign and malignant diseases of the liver, pancreas and bile ducts. In laparoscopic surgery, there is a need for accurate descriptions of liver vascularization to avoid iatrogenic vascular changes [25, 26, 30].

An understanding of the extent of vascularization by the IPA is significant because, apart from the main supply of the diaphragm, it can form collateral circulation. Liver cancers commonly derive their arterial supply from the hepatic arteries, and the RIPA and the LIPA are pathways extrahepatic collateral arteries; they can also supply hepatic malignancies, because they neighbour hepatic segments as they transverse the bare area of the liver [11, 13]. Among the arterial pathways that provide liver cancer, both RIPA and LIPA represent nearly half of the collaterals, with RIPA being the most common [11, 13] and LIPA being the fourth or sixth most common [11, 13]. As a result, both RIPA and LIPA are used during transcatheter arterial chemoembolization of liver tumours, especially those located in the peripheral segments of the liver (1–4) [11, 13].

Variations in the origin of the phrenic arteries are numerous and supplementary phrenic vessels are common [1, 10, 15, 29, 32, 33]. The inferior phrenic arteries arise from a common trunk (55%), from the aorta or the celiac trunk in 18–30% of cases, or as independent branches from these same sources in 62% [32]. Other sources may be the hepatic, left gastric, renal, suprarenal, or superior mesenteric arteries in about 8% of cases [32]. When independent, the right and left phrenics usually arise asymmetrically. The study by Grieg et al. [10] of 848 bodies, the origin of the inferior phrenic is as follows: right and left separately from the celiac trunk, 20.3%; as a common trunk from the aorta, 19.7%; right artery from the aorta, left from the celiac, 14.2%; common trunk from the celiac, 13.6%; separately from the aorta, 13.2%; right from the celiac, left from the aorta, 6.8%; right from the renal, left from the aorta, 3.7%; right from the renal, left from the celiac, 3.5%; right and left from the left gastric, 0.7%; right from left gastric, left from aorta. 0.5%; right from the aorta, left from renal, 0.5%; right from celiac, left from left gastric, 0.5%; right from aorta, left from left gastric, 0.4%; right and left from renal, 0.4%; and all other sources and combinations, 1.9% [10]. Also, in some cases in which the right inferior gastric arose from a renal artery, it was from a superior polar renal artery [10].

More recent studies indicate that the RIPA most commonly originates from the abdominal aorta, as noted by Kimura et al. [14], Basilie et al. [5] and Gürses et al. [11]; however, Loukas et al. [16] and Aslaner et al. [4] report the most common origin of RIPA to be the celiac trunk. The second most frequent type of RIPA origin was found to be the celiac trunk by Kimura et al. [14] and Basilie et al. [5], the abdominal aorta by Loukas et al. [16] and Aslaner et al. [4], and the right renal artery by Gürses et al. [11]. It is worth mentioning that Kimura et al. [14] report the possibility of RIPA originating from the dorsal pancreatic artery, while Aslaner et al. [4] note the possibility of it originating from the common hepatic artery. The difference between the types of origin of the right inferior phrenic artery are shown in Table 1.

**Table 1:** Comparison types of origin of the right inferior phrenic artery

Types of origin of the RIPA	Loukas et al. (%)	Kimura et al. (%)	Basilie et al. (%)	Gürses et al. (%)	Aslaner et al. (%)	Present case
Abdominal aorta	38	57	49	50	25.2	–
Celiac trunk	40	30	41	3.85	30.7	–
Right renal artery	17	11	5.5	7.69	10.4	–
Proper hepatic artery	2	–	0.5	–	–	–
Left gastric artery	3	2	4	3.85	4.1	–
Dorsal pancreatic artery	–	1	–	–	–	–
Common hepatic artery	–	–	–	–	0.1	–
The common trunk of the RIPA, SSA and ISA from the RA	–	–	–	–	–	100

*RIPA* right inferior phrenic artery, *SSA* superior suprarenal artery, *ISA* inferior suprarenal artery, *RA* renal artery

The suprarenal arteries demonstrate great morphological variety [2, 7–9, 20, 21]. Dutta et al. [8] note that the SSA did not have any variations in origin; this contrasts with our current report, where the SSA origin of the common trunk which originates from the right renal artery. Dutta et al. [8] report the MSA to be absent from the right side in 29% of cases; however, our present findings do not indicate the presence of MSA. While Dutta et al. [8] report variability in the origin of the ISA, i.e. 18% originated from the gonadal arteries and 6% from the right lateral margin of the abdominal aorta, our present study found the ISA to originate from the common trunk.

In a study of 200 cadaveric dissections, Michels [22, 23] describes ten types of hepatic arterial variant. The full classification with its frequency of occurrence is as follows: Type I (normal pattern) − 81%; Type II (a replaced LHA from the left gastric artery) 3%; Type III (a replaced RHA from the superior mesenteric artery) 3.7%; Type IV (replaced RHA and LHA) 0.8%; Type V (an accessory LHA) 3.2%; Type VI (an accessory RHA) 1.6%; Type VII (accessory LHA and RHA) 0.2%; Type VIII (a replaced LHA or RHA with other hepatic artery being an accessory one) 0.35%; Type IX (the hepatic trunk as a branch of the superior mesenteric artery) 1.2% and Type X (common hepatic artery branched from the left gastric artery) 0.04%.

This classification by Michels was later modified by Hiatt et al. [12]. In a study of 1000 livers, Hiatt et al. [12] note the presence of a normal hepatic artery in 757 specimens (75.7%), a LAHA originating from the left gastric artery in 97 specimens (9.7%) and an RAHA originating from the superior mesenteric artery in 106 specimens (10.6%). In 23 cases they also report the presence of a double-replaced pattern, where the right hepatic artery was a branch of the superior mesenteric artery and the left hepatic artery was a branch of the left gastric artery [12]. They also note two variants of origin of the common hepatic artery: as a branch of the superior mesenteric artery in 15 cases (2.3%) and originating directly from the aorta in two cases (1.5%).

López-Andújar et al. [17] classified 12 types of hepatic arterial variations. The full classification is as follows: Type 1 (normal hepatic arterial) 70%; Type 2 (a replaced left hepatic artery arises from the left gastric artery) 9.7%; Type 3 (a replaced right hepatic artery arises from the superior mesenteric artery) 7.8%; Type 4 (presence both replaced right and left hepatic arteries, and the replaced right hepatic artery originated from the superior mesenteric artery, while the left originates from the left gastric artery) 3.1%; Type 5 (the LAHA arises from the left gastric artery) 3.9%; Type 6 (the RAHA originated from the superior mesenteric artery) 0.6%; Type 7 (the LAHA originated from the left gastric artery and RAHA arises from the superior mesenteric artery) 0.6%; Type 8 (replaced left hepatic artery arises from the left gastric artery and the RAHA originated from the superior mesenteric artery) 0.3%; Type 9 (the common hepatic artery arises from the superior mesenteric artery) 2.5%; Type 10 (the common hepatic artery originates from the left gastric artery) 0%; Type 11 (the common hepatic artery arises from the superior mesenteric artery and LAHA is a branch of the left gastric artery) 0.3%; Type 12 (the common hepatic artery arises directly from the aorta) 0.7%.

Noussios et al. [24] report the presence of a normal hepatic arterial anatomy in 81% of examined cases, and 19% were variations on the hepatic arteries: the LAHA was present in 1.6%, while the RAHA in 3.2%; replaced right hepatic artery originated from the superior mesenteric artery in 3.7%, while a replaced left hepatic artery originated from the left gastric artery in 3% of cases. Both replaced right and left hepatic artery were observed in 0.8% of cases [24]. Our previous studies also describe the existence of accessory hepatic arteries, namely five RAHA arising from the celiac trunk, one RAHA branching off the proper hepatic artery, and one RAHA originating from the superior mesenteric artery; it also describes a variant where the LAHA arises from the left gastric artery [25].

Some variants of the AHA were also described as a case report. Bastos-Neves et al. [6] reported a rare anatomical variation of the hepatic arterial supply: a RAHA arising directly from the celiac trunk. Panagouli et al. [27] describe a RAHA originating from the left gastric artery, while Polguj et al. [30] describe a RAHA originating from the common hepatic artery near the celiac trunk, which ran behind the portal vein to the right lobe of the liver. Yamashita et al. [35] describe a RAHA branching from the gastroduodenal artery. Lurie et al. [18] and Paraskevas et al. [28] describe a LAHA originating from the left gastric artery.

The identification of variations and anomalies in the right hepatic artery is not only of value to the anatomist, but also to the surgeon. The RAHA may be injured during resection of the pancreatic head, because the artery is located in close proximity to the portal vein [34]. The presence of replaced right hepatic artery can save live of patients with biliary tract cancer, because they are further away from the bile duct and tendo to save a cancer, allowing the tumour to be excised [34].

The common trunk and the branches off this trunk described in this case report are clinically important: the right inferior phrenic artery may form collateral pathways for liver cancer, thus better facilitating surgical procedures. The presence of the AHA is an important consideration in surgery, because recent literature suggests livers with an AHA are preferred for donor liver transplantation. In addition, Aramaki et al. [3] propose the use of right-sided grafts from donors with an LAHA and a left-sided graft from donors with an RAHA. Knowledge of such anatomical variation is clearly significant in these cases.

## Conclusion

In conclusion, although the common trunk of the inferior phrenic artery, superior and inferior suprarenal artery and presence of the right accessory hepatic artery observed in this case are very rare, it might be a highly significant factor in the arterial supply to this region. Preoperative knowledge of such anatomic variants is essential in planning surgical procedure such as liver transplantation or removal of the liver lobe.
